# Acupoint catgut embedding for patients with migraine: A protocol for systematic review

**DOI:** 10.1097/MD.0000000000021268

**Published:** 2020-07-31

**Authors:** Jie Li, Feng Zhang, Wenchun Wang, Rizhao Pang, Jiancheng Liu, Qiuhong Man, Anren Zhang

**Affiliations:** a School of Acupuncture-Moxibustion and Tuina, Chengdu University of Traditional Chinese Medicine; b The General Hospital of Western Theater Command, Chengdu; c Department of Clinical Laboratory; d Department of Rehabilitation Medicine, Shanghai Fourth People's Hospital Affiliated to Tongji University School of Medicine, Shanghai, China.

**Keywords:** acupoint catgut embedding, migraine, protocol, systematic review

## Abstract

**Background:**

Nowadays, acupoint catgut embedding is being used widely in the treatment of migraine. So far, there is no a systematic review has been conducted. Therefore, the purpose of this paper is to systematically evaluate the efficacy and safety of acupoint catgut embedding on migraine.

**Methods:**

We will search the following databases from their inception to May 2020: PubMed, Embase, Medline, EBSCO, Web of Science, Chinese National Knowledge Infrastructure, Chinese Biomedical Literature Database, Wan Fang Database, the Chongqing VIP Chinese Science and Technology Periodical Database, Cochrane Library. In addition, we will manually retrieve other resources including conference articles, and gray literature. The randomized controlled trials in English or Chinese associated with acupoint catgut embedding for migraines will be included. The data collection and analysis will be conducted independently by 2 reviewers. Meta-analysis will be performed using Rev Man V.5.3.5 statistical software.

**Results:**

This study will provide a high-quality synthesis to evaluate the efficacy and safety of acupoint catgut embedding for patients with migraine.

**Conclusion:**

This systematic review will provide evidence to judge whether acupoint catgut embedding is an effective and safe intervention for patients with migraines. It will provide reliable evidence for its extensive application.

**OSF Registration number:**

DOI 10.17605/OSF.IO/RP9NW.

## 1 Introduction

About 11.6% and a lifetime prevalence of 15% of people suffer from migraines.^[1]^ A prevalence of 15% has been reported in the United States and about 10% in Asia.^[2,3]^ Migraine is a common refractory disease with high social and economic impact.^[4–6]^ According to the World Health Organization, it has been classified as one of the most serious and chronic diseases.^[7]^ Although some drugs (topiramate, divalproex sodium, metoprolol) can be used to reduce the frequency of migraine attacks, patients can experience adverse reactions. In addition, data from clinical trials study show a high drop-out rate, which indicate that migraine sufferers do not receive the drug.^[8]^

Acupuncture is an important part of complementary and alternative medicine. In recent years, the efficacy of acupuncture has been proven, and it has been widely used in the treatment of migraine due to almost no side effects.^[9–12]^ However, the acupuncture treatment cycle of migraine is too long, which brings great inconvenience to the frequent application of acupuncture. Such as the need to receive 3 to 5 treatments per week, and it takes a lot of time each time. During the treatment period, patients need to maintain a posture for a long time. Acupoint catgut embedding is a kind of modern acupuncture. It needs to the use of sterile forceps to put 3-0 catgut (1–1.5 cm) into the needle tip of No. 9 disposable sterile needles, the catgut is parallel with the inner edge of the needle tip, and the needle is followed by a blunt acupuncture needle, inserting the sterile needle into the disinfected acupuncture points. After getting de-qi, the acupuncture needle is pushed slowly.^[13,14]^

Acupoint catgut embedding combines the advantages of acupuncture, point acupuncture, and catgut.^[15,16]^ Because its sustainable and curative effect can last 15 to 20 days, it makes up for the shortcomings of acupuncture therapy. Such as long treatment cycle, frequent frequency, and short stimulation time. In addition, acupoint catgut embedding is significantly better than traditional acupuncture in the way of reducing the treatment cost and time.^[17–19]^ At present, more and more acupoint catgut embedding is being used in the treatment of migraine. However, there is no systematic review at home and abroad to evaluate the efficacy and safety of acupoint catgut embedding in the treatment of migraine. Therefore, this review will assess the efficacy and safety of acupoint catgut embedding therapy for headache compared with western medicine and acupuncture. This systematic review will be the first to evaluate the effects of acupoint catgut embedding on migraines, and I hope we can provide convincing results.

## 2 Methods and analysis

### 2.1 Eligibility criteria

#### 2.1.1 Types of studies

The types of studies including randomized controlled trials (RCTs) and quasi-RCTs.

#### 2.1.2 Types of participants

The types of participants including who have been diagnosed with migraines, according to the definition of the Headache Classification Subcommittee of the International Headache Society. People who have been diagnosed with migraines include regardless of their age, sex, or race.

#### 2.1.3 Types of intervention

Experimental intervention measures should be acupoint catgut embedding alone. The control group include drugs, body acupuncture therapy, or shame acupoint catgut embedding.

#### 2.1.4 Types of outcome measures

The primary outcome will be the overall effective rate.

The secondary Outcome Measures are the differences in frequency of migraine attacks, average duration of a migraine attack, rate of rescue medication used, Migraine-Specific Quality-of-Life Questionnaire before randomization. Other outcomes included safety and adverse events of catgut embedding alone will be observed.

### 2.2 Search methods for identification of studies

We will search the following databases from their inception to May 2020: PubMed, Embase, Medline, Chinese National Knowledge Infrastructure, Chinese Biomedical Literature Database, the Chongqing VIP Chinese Science and Technology Periodical Database, EBSCO, Web of Science, Wan Fang Database, the Chongqing VIP Chinese Science and Cochrane Library. In addition, we will manually retrieve other resources including conference articles, and gray literature. The RCTs in English or Chinese associated with acupoint catgut embedding for migraines will be included. The example search strategy for Pubmed in Table 1 will be used. This search strategy will be used in several other databases.

**Table 1:**
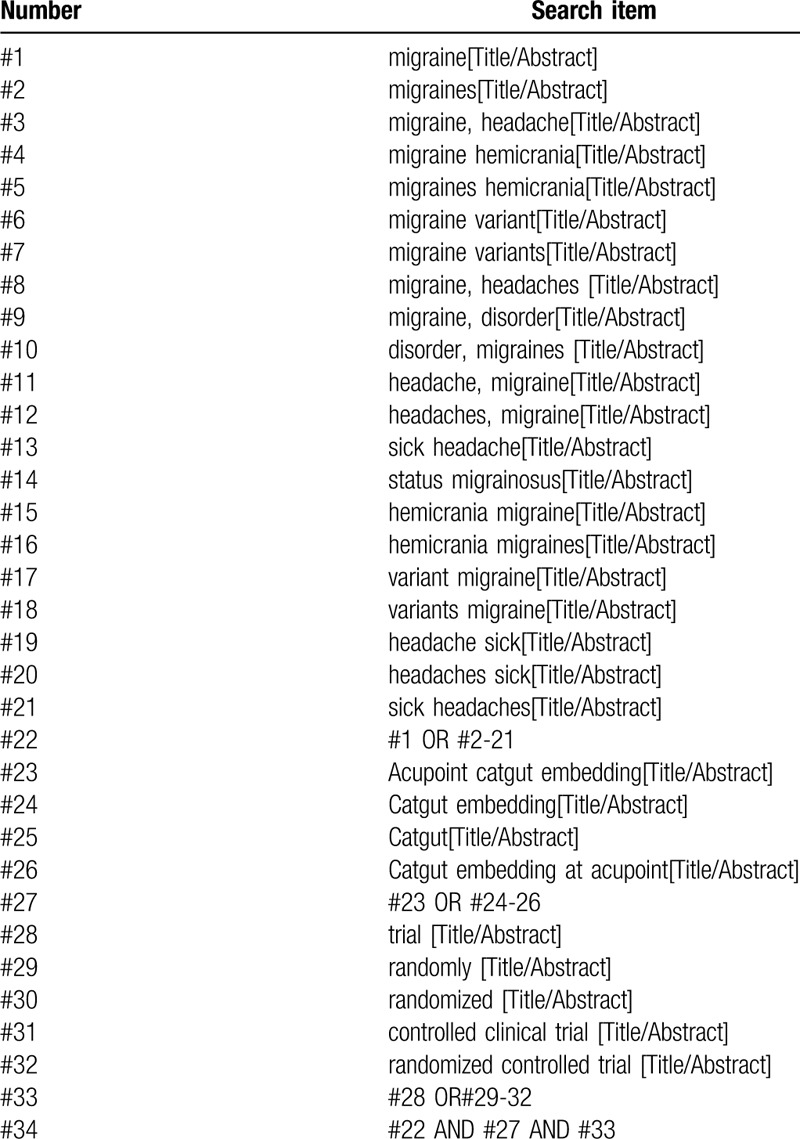
PubMed search strategy draft.

### 2.3 Data collection and analysis

#### 2.3.1 Data extraction and management

**Figure 1:**
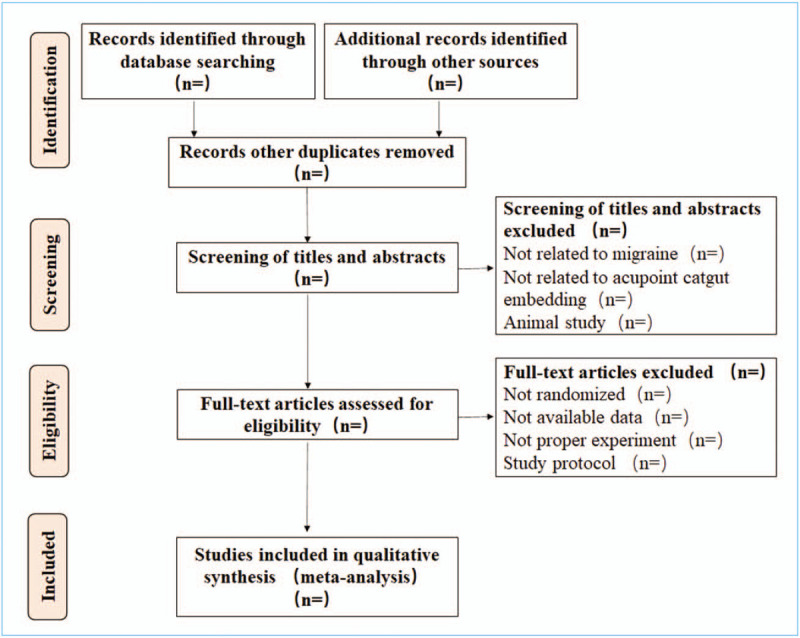
Flow chart of study selection.

After finishing the search work, the screening process will be completed independently by 2 reviewers (LJ and ZF). After reading titles, abstracts, and full texts. The third reviewer (ZAR) will evaluate whether the studies will be satisfied according to inclusion criteria. The selection process is shown in Figure 1 below.

#### 2.3.2 Assessment of risk of bias

Two independent reviewers (LJ and LJC) will use the bias tool of Cochrane Manual V.5.1.0 to conduct the assessment of risk about bias. The process includes: random sequence generation, blinding, allocation sequence concealment, incomplete data, selective reporting, and other sources. Any assessment of the bias has caused controversy will be resolved by the third reviewer (WWC). The biased results will be divided into 3 levels: “low risk of bias” “high risk of bias,” and “uncertain risk of bias.”

#### 2.3.3 Assessment of heterogeneity

We will calculate the value of the I^2^ statistic to test the heterogeneity of data. When the *I*^2^ value exceeds 50%, the trials statistical heterogeneity is significant, and our meta-analysis will not be conducted. At same time, we carry out subgroup stratification analysis to explore the possible reasons of causing heterogeneity.

#### 2.3.4 Unit of analysis issues

The unit of analysis will be conducted by the independent reviewers (PRZ).

#### 2.3.5 Dealing with missing data

Any missing data will be complemented by the independent reviewers (PRZ) through contacting with the corresponding author.

#### 2.3.6 Assessment of reporting biases

The funnel charts will be used to assess reporting biases. When the numbers of available studies are sufficient, we will conduct a test for funnel plot asymmetry using the Egger method.

#### 2.3.7 Data synthesis

We will use the RevMan V.5.3 to analyze the data. The test result indicated little or no heterogeneity. The specific methods are as follows: The fixed-effects model will be used for data synthesis when the *I*^2^ test is less than 50%. The random-effects model will be conducted for data synthesis when the *I*^2^ test is between 50% and 75%. The meta-analysis will not be performed when the *I*^2^ test is higher than 75%. When data cannot be synthesized, we will try to explore the possible reasons, and provide a descriptive analysis to solve this problem.

#### 2.3.8 Subgroup analysis

We will use Subgroup analysis to evaluate high heterogeneity. The factors affecting the heterogeneity include the different combinations of acupoint catgut embedding, different course time, and other factors.

#### 2.3.9 Sensitivity analysis

We will use sensitivity analysis to test the robustness of the main decisions in the review process, which including the impact of quality of methods, sample size, and related issues on the study. In order to screen more inferior quality studies and quantify statistical heterogeneity, the meta-analysis and *I*^2^ value will be reused.

#### 2.3.10 Grading the quality of evidence

The Grading of Recommendations Assessment, Development, and Evaluation approach will be used to evaluate the quality of evidence for all results of this systematic review. The quality will be divided into 4 levels: high, moderate, low, or very low.^[20]^

## 3 Discussion

Migraine is not only a common refractory disease, but also a disabling neurological disease.^[6,7]^ Currently, some western medicines on the market can be used to reduce the frequency of migraine attacks, but they always can cause adverse reactions in patients. Some clinical trial data show poor adherence to these medications in migraine patients.^[8]^ In recent years, with the development of complementary and alternative medicine, acupuncture has been widely used in the treatment of migraine.^[12,21]^ However, acupuncture therapy has the characteristics of long cycle, high application frequency, short stimulation time, much time and much money, especially for the treatment of migraine. In this case, Acupoint catgut embedding, as a representative of the combination of modern acupuncture and traditional acupuncture, is widely used. It is characterized by long and sustainable effect, simple operation, saving time, low cost, which compensate for the shortcomings of acupuncture.^[16,22]^ Although the advantages of acupoint catgut embedding in the treatment of migraine are obvious, there is still no systematic review in English at present.

This article will be the first review on the systematic evaluation of acupoint catgut embedding for migraine. It will draw reasonable conclusions by collecting evidence, sorting out data and analyzing data about the efficacy and safety of acupoint catgut embedding in the treatment of migraine. We hope this study will provide convincing evidence for both patients and clinicians. However, this systematic review has some limitations. The literature collected in this study will be published in English and Chinese, and no articles in other languages are collected. In addition, there may not be much literature on acupoint catgut embedding for the treatment of migraine.

## Author contributions

**Data collection:** Jie Li, Feng Zhang.

**Formal analysis:** Wenchun Wang, Jie Li.

**Funding acquisition:** Anren Zhang.

**Investigation:** Anren Zhang, Qiuhong Man, Wenchun Wang.

**Software application:** Jiancheng Liu.

**Supervision:** Qiuhong Man, Anren Zhang.

**Writing – original draft:** Jie Li.

**Writing – review & editing:** Rizhao Pang.

## References

[1] Burch RC Loder S Loder E The prevalence and burden of migraine and severe headache in the United States: updated statistics from government health surveillance studies. Headache 2015;55:21–34. 25600719 10.1111/head.12482

[2] Wang SJ Epidemiology of migraine and other types of headache in Asia. Curr Neurol Neurosci Rep 2003;3:104–8. 12583837 10.1007/s11910-003-0060-7

[3] Chen J Zhao L Zheng H Evaluating the prophylaxis and long-term effectiveness of acupuncture for migraine without aura: study protocol for a randomized controlled trial. Trials 2013;14:361. 24171782 10.1186/1745-6215-14-361 PMC3816544

[4] Lipton RB Scher AI Steiner TJ Patterns of health care utilization for migraine in England and in the United States. Neurology 2003;60:441–8. 12578925 10.1212/wnl.60.3.441

[5] Diamond M Dahlöf C Papadopoulos G Topiramate improves health-related quality of life when used to prevent migraine. Headache 2005;45:1023–30. 16109116 10.1111/j.1526-4610.2005.05183.x

[6] Goadsby P Lipton R Ferrari M Migraine - current understanding and treatment. N Engl J Med 2002;346:257–70. 11807151 10.1056/NEJMra010917

[7] Menken M Munsat T Toole J The global burden of disease study: implications for neurology. Arch Neurol 2000;57:418–20. 10714674 10.1001/archneur.57.3.418

[8] Linde M Mulleners WM Chronicle EP Valproate (valproic acid or sodium valproate or a combination of the two) for the prophylaxis of episodic migraine in adults. Cochrane Database Syst Rev 2013;6:CD010611. 10.1002/14651858.CD010611 PMC10373438 23797677

[9] Zhao L Chen J Li Y The long-term effect of acupuncture for migraine prophylaxis: a randomized clinical trial. JAMA Intern Med 2017;177:508–15. 28241154 10.1001/jamainternmed.2016.9378

[10] Wells RE Bertisch SM Buettner C Complementary and alternative medicine use among adults with migraines/severe headaches. Headache 2011;51:1087–97. 21649654 10.1111/j.1526-4610.2011.01917.x PMC3627391

[11] Kristoffersen ES Grande RB Aaseth K Management of primary chronic headache in the general population: the Akershus study of chronic headache. J Headache Pain 2012;13:113–20. 21993986 10.1007/s10194-011-0391-8 PMC3274574

[12] Li Y Liang F Yang X Acupuncture for treating acute attacks of migraine: a randomized controlled trial. Headache 2009;49:805–16. 19438740 10.1111/j.1526-4610.2009.01424.x

[13] Ren XY Study on the source and mechanism of suture at acupoint catgut embedding [Chinese]. China J Tradit Chin Med Pharm 2004;19:757–9.

[14] Xu MZ Clinical observation on 100 cases of obesity treated with acupoint catgut embedding [Chinese]. Chin Acupunct Moxibustion 2002;22:24–5.

[15] Wang HR Yin LL Li CM Advances in clinical and mechanisms of acupoint catgut embedding therapy for obesity [Chinese]. Shaanxi Chin Med 2011;32:374–6.

[16] Ren XY Study on the source and mechanism of suture at acupoint catgut embedding [in Chinese]. China J Tradit Chin Med Pharm 2004;19:757–9.

[17] Huang C Pan W Comparation of effect and cost-benefit analysis between acupoint catgut-embedding and electroacupuncture on simple obesity [Chinese]. Zhongguo Zhen Jiu 2011;31:883–6. 22043672

[18] Zhao YJ Ma HY Cai DG Progress in research on the clinical and mechanism of acupoint suture and acupoint injection [in Chinese]. Mod J Integr Tradit Chin West Med 2013;22:784–7.

[19] Gao DR Gao DG Gao L Observation on the curative effect of acupoint catgut embedding in treating rheumatoid arthritis [Chinese]. Shanghai J Acupunct Moxibustion 2007;26:27.

[20] van de Griendt EJ Tuut MK de Groot H Applicability of evidence from previous systematic reviews on immunotherapy in current practice of childhood asthma treatment: a GRADE (Grading of Recommendations Assessment, Development and Evaluation) systematic review. BMJ Open 2017;7:e016326. 10.1136/bmjopen-2017-016326 PMC5770836 29288175

[21] Bauer BA Tilburt JC Sood A Complementary and alternative medicine therapies for chronic pain. Chin J Integr Med 2016;22:403–11. 27339090 10.1007/s11655-016-2258-y

[22] Bu DEP Zhang XM Treatment principle and clinical value of acupoint catgut embedding therapy. Med J West China (China) 2009;21:852–4.

